# Six weeks' sebacic acid supplementation improves fasting plasma glucose, HbA1c and glucose tolerance in db/db mice

**DOI:** 10.1111/j.1463-1326.2010.01308.x

**Published:** 2010-12

**Authors:** M Membrez, C J Chou, F Raymond, R Mansourian, M Moser, I Monnard, C Ammon-Zufferey, K Mace, G Mingrone, C Binnert

**Affiliations:** 1 Nestlé Research CenterRoute du Jorat, Lausanne, Switzerland; 2 Department of Internal Medicine, Catholic University of RomaRome, Italy

**Keywords:** glucose metabolism, liver, type 2 diabetes

## Abstract

**Aim:** To investigate the impact of chronic ingestion of sebacic acid (SA), a 10-carbon medium-chain dicarboxylic acid, on glycaemic control in a mouse model of type 2 diabetes (T2D).

**Methods:** Three groups of 15 db/db mice were fed for 6 weeks either a chow diet (Ctrl) or a chow diet supplemented with 1.5 or 15% (SA_1.5%_ and SA_15%_, respectively) energy from SA. Fasting glycaemia was measured once a week and HbA1c before and after supplementation. An oral glucose tolerance test (OGTT) was performed at the end of the supplementation. Gene expression was determined by transcriptomic analysis on the liver of the Ctrl and SA_15%_ groups.

**Results:** After 42 days of supplementation, fasting glycaemia and HbA1c were ∼70 and 25% lower in the SA_15%_ group compared with the other groups showing a beneficial effect of SA on hyperglycaemia. During OGTT, plasma glucose area under the curve was reduced after SA_15%_ compared with the other groups. This effect was associated with a tendency for an improved insulin response. In the liver, Pck1 and FBP mRNA were statistically decreased in the SA_15%_ compared with Ctrl suggesting a reduced hepatic glucose output induced by SA.

**Conclusion:** Dietary supplementation of SA largely improves glycaemic control in a mouse model of T2D. This beneficial effect may be due to (i) an improved glucose-induced insulin secretion and (ii) a reduced hepatic glucose output.

## Introduction

Insulin resistance is the hallmark of type 2 diabetes (T2D) and leads to the inability for the body to utilize glucose properly. In diabetic patients, fasting glycaemia is increased and in the postprandial state, insulin fails to completely suppress hepatic glucose production (HGP). It is well established that increased HGP, rather than decreased peripheral glucose uptake is responsible for fasting hyperglycaemia [1–4]. Even though the relative contribution of gluconeogenesis (GNG) and glycogenolysis to HGP is debated in T2D, it is established that GNG contributes to hyperglycaemia both in fasting and postprandial states.

Sebacic acid (SA) is a 10-carbon medium-chain dicarboxylic acid (MCDA) chemically very close to its monocarboxylic medium-chain lipid counterpart (decanoÏc acid). Its caloric content is 6.6 kcal/g and it seems to behave metabolically like a lipid: once in the body, MCDA is, either partially *β*-oxidized to suberic and adipic acids in the peroxisomes and/or to succinic acid in the mitochondria [5,6] or directly excreted into the urine [7,8]. SA is very rarely ingested as such because barely available in usual food but present in low amounts in honey [9] and concentrations of MCDA can be increased via *ω*-oxidation of ingested medium-chain *mono*carboxylic acids [10–12]. Even though sebacic and dodecanedioic acids (the dicarboxylic acid with 12 carbons) are not classified as classical nutrients, they have been proposed as an alternative substrate to glucose for critically ill patients and T2D because MCDA provide rapidly available energy [13,14]. More recently, SA intake in a mixed meal has been shown to decrease postprandial glycaemia and hepatic glucose output in T2D patients [15]. Nevertheless, the mid- and long-term benefits of oral SA intake on glycaemic control are unknown. This study was therefore conducted in order to evaluate the possible antidiabetic properties of supplemented SA and to spread light into its mechanism of action. We compared the impact of two doses of SA supplementation on fasting glycaemia, HbA1c, glucose tolerance and liver gene expression in a mouse model of T2D (db/db BKS mouse) after 6 weeks of supplementation.

## Research Design and Methods

### Animals, Diet and SA Supplementation

Forty-five 6–8 week old male BKS.Cg-m+/+Leprdb/J *db*/*db* mice (Charles River Laboratories, France) were housed individually. After 2 weeks of habituation, mice were randomized into three groups (n = 15/group) based on their (i) basal plasma glucose levels after a 6-h fasting period and (ii) body weight. Differences between groups' median were smaller than 1.2 mmol/l (8%) for glycaemia and 0.5 g (1%) for body weight. The control group mice were given free access to a chow diet (diet 3437, Kliba Nafag, Basel, Switzerland: ∼31, 11 and 58% of energy from protein, fat and carbohydrate, respectively); 7.76 g/kg food (∼1 g/kg body weight/day) of SA were added in the chow food for the second group (SA_1.5%_), corresponding to 1.5% supplementary energy coming from SA. The third group (SA_15%_) received the chow diet where 77.6 g/kg food SA were added (∼10 g/kg body weight/day), corresponding to 15% energy coming from SA. All animals had free access to water and food all along the study.

SA was bought from Hengshui Dongfeng Chemical (HengShui City, Hebei Province, China) and its purity was 100 ± 0.5%.

Throughout the 42-day supplementation period, body weight, water and food intake were recorded weekly. Body composition of each animal was measured in a conscious state on days −1 and 31 using an EchoMRI™ 3-in-1 (Echo Medical Systems, Houston, TX, USA). Six hours fasting glycaemia was measured weekly and insulin concentrations were measured at days 1, 14 and 42 during supplementation period. Oral glucose tolerance tests (OGTTs) were conducted in overnight fasted (15 h) mice on day 36. On day 42, 6 h fasted mice were killed for tissue collection.

Livers were collected immediately after exsanguination by portal puncture and flash frozen in liquid nitrogen. All procedures were approved by the cantonal veterinary authorities (Lausanne, Switzerland) under authorization number 2191.

### Oral Glucose Tolerance Test

OGTT was performed at the end of the supplementation period. Mice were fasted for 15 h starting at 17:00 hours. After measuring fasting glucose concentration in blood obtained by tail incision, using an Ascensia Elite XL glucometer (Bayer AG, Zurich, Switzerland), animals were given a glucose solution at 1.5 g/kg (wt/BW) by oral gavage at time 0. Blood glucose was measured after 15, 30, 60 and 120 min. Blood was also collected in EDTA coated tubes at 0, 15 and 60 min for insulin analyses.

### Liver Biochemistry

Total lipids were extracted from 200 mg frozen liver according to Folch et al. [16]. Triglycerides (TG) were first hydrolysed in a basic solution (0.5 N KOH in ethanol) and then measured using a commercial enzymatic triglyceride analysis kit (PAP 150, BioMérieux, Marcy l’Etoile, France). Total cholesterol (Roche Diagnostics, Basel, Switzerland) was measured following manufacturer's instructions. For glycogen, flash frozen liver (50–100 mg) was incubated in 2 ml 30% KOH for 15–30 min in a boiling water bath. After homogenization, glycogen was precipitated with 3 ml 96% ethanol followed by a centrifugation at 10 000 *g* for 10 min. Pellets were resuspended in 1 ml distilled water for the amyloglucosidase digestion. Samples (200 µl) were incubated in 1.8 ml of lyophilized amyloglucosidase dissolved in acetate buffer (0.2 M, pH 4.8 at a final concentration of 10 U/ml) for 2 h at 40 °C. The resulting glucose solution was then measured with a Quantichrom Glucose Assay Kit (BioAssay Systems, Hayward, CA, USA).

### Plasma Biochemistry

Plasma triglyceride (PAP 150, BioMérieux), non-esterified fatty acids (FAs) (Wako, Neuss, Germany), insulin (IBL, Hamburg, Germany) and adiponectin (Millipore Corporation, Billerica, MA, USA) levels were measured using commercial kits. Plasma alanine (#DF43A) and aspartate (#DF41A) transaminases activity were measured using the Dimension Xpand Plus (Siemens HealthCare Diagnostics, Deerfield, IL, USA). HbA1c (#DF105) percentage was determined using the Dimension Xpand Plus (Siemens HealthCare Diagnostics) with 100 µl of fresh total blood.

### Gene Expression Analysis

#### Total RNA Extraction, Labelled-cRNA Synthesis and Hybridization

Liver tissue samples (∼10 mg wet weight) were disrupted and homogenized in lysis buffer using a FastPrep instrument and lysing tubes containing ceramic beads (MP Biomedicals, Irvine, CA, USA). Total RNA was then extracted and purified with the RNAdvance Tissue Kit (Agencourt, Beverly, MA, USA) through an automated procedure on a robotic station. After extraction, RNA quality was checked for RNA integrity numbers ≥8 by Agilent 2100 Bioanalyser (Agilent Technologies, Palo Alto, CA, USA). All cRNA targets were synthesized, labelled and purified according to the Illumina TotalPrep RNA amplification protocol (Applied Biosystems/Ambion, Austin, TX, USA) through an automated procedure originally deployed for Affymetrix sample preparation that was adapted for Illumina [17]. Briefly, 200 ng of total RNA was used to produce double-stranded cDNA, followed by an *in vitro* transcription, and by cRNA labelling with biotin. This method is based on the Eberwine T7 procedure [18]. Prior to the hybridization on the arrays, 750 ng of biotin labelled cRNAs was added to the hybridization mix, which contained control oligonucleotides (such as negative and hybridization controls), hybridization buffer and water. Then, 15 µl of each hybridization mix was dispensed on the arrays. After an overnight hybridization (16 h, 58 °C), the arrays were washed to remove non-hybridized material, and stained with Streptavidin–Cy3 which bind to biotin. All samples were analysed with MouseRef-8 v1.1 Expression BeadChips (Illumina, San Diego, CA, USA), which interrogate 24 600 transcripts.

#### Microarray Processing and Data Acquisition

Scanning was performed using the BeadArray Reader (Illumina), which provides intensity values for all transcripts, measuring the signal emitted by the Streptavidin–Cy3 conjugates responding to a laser excitation. Signal intensities were extracted and summarized in the BeadStudio software (Illumina). Raw data were expressed as absolute signal intensities.

### Statistical Analysis

Data are presented as median ± standard error of the median (s.e.median). The s.e.median was computed based on the robust standard deviation: Sn of Rousseeuw, p values were corrected for multiplicity of test (Sidak–Bonferroni procedure). Insulinaemia, HbA1c, other plasma parameters, OGTT [time 0 and area under the curve (AUC)], liver parameters, NMR data were analysed with non-parametric tests, that is, Wilcoxon tests (estimated difference by Hodges–Lehmann). The change on HbA1c is tested against 0 for each group by Wilcoxon signed-rank tests. The software R 2.6.1 was used to perform the analysis [19]. For mRNA expression, quality control and statistical analysis of microarray data were carried out with Partek software (Partek, St. Louis, MO, USA). After quantile normalization and a log2 transformation, quality control of the data was performed with a Pearson correlation matrix and a principal component analysis on all probes to help determine possible outliers. To assess which transcripts were differentially expressed between SA_15%_ and control groups, a one-way analysis of variance (ANOVA) was performed, followed by a global error assessment (GEA) [20]. The GEA results in a robust mean squared error (MSE), which replaces the current MSE from the classical ANOVA, a new *F* statistic is recalculated and a robust p value derived.

## Results

### Body Weight and Body Composition

After the end of the supplementation, neither body weight (44.4 ± 0.9, 44.1 ± 1.3 and 43.5 ± 0.7 g) nor % body fat (58.4 ± 0.7, 59.7 ± 0.9 and 58.7 ± 0.6% BW) was different among groups for the Ctrl, SA_1.5%_ and SA_15%_ groups, respectively.

### Food and Water Intake

Cumulated food intake over the 42 days was reduced (p < 0.05) in the SA_15%_ group compared to others (513.4 ± 13, 611.3 ± 15.6 and 582.1 ± 12.2 kcal, for the SA_15%_, Ctrl and SA_1.5%_ groups, respectively). At day 36, water intake was higher (p < 0.001) in the Ctrl (11.9 ± 1.2 ml) and SA_1.5%_ (10.2 ± 1.5 ml) than in the SA_15%_ group (4.0 ± 0.4 ml).

### Fasting Glycaemia, Insulinaemia and HbA1c

**Figure 1:**
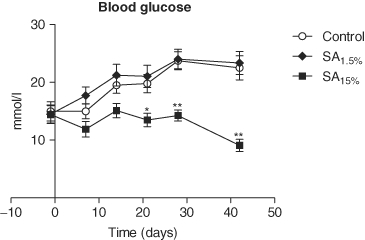
Fasting blood glucose measured weekly during the study after a 6-h food deprivation. Open circle: Ctrl, black diamond: SA_1.5%_ and black square: SA_15%_. ^*^p value < 0.05, ^**^p value < 0.01 as compared to control group.

Glycaemia evolution during supplementation is depicted in figure 1. We observed a significant increase in glycaemia over the 6 weeks' period of the trial in the non-supplemented and SA_1.5%_ groups. On the opposite, in the SA_15%_ group, fasting glycaemia plateaued at a value very close to the presupplementation values, leading to a 40% difference compared to the non-supplemented and the SA_1.5%_ group at day 42 (p < 0.01). Fasting plasma insulin concentrations were reduced during the course of the trial without any differences between groups: for the Ctrl, SA_1.5%_ and SA_15%_ groups it decreased from 982 ± 167, 1148 ± 148 and 918 ± 121 pm/l before supplementation to 145 ± 44, 184 ± 35 and 173 ± 43 pm/l after supplementation, respectively. The long-term glucose control was also evaluated by measuring HbA1c (Table 2). Briefly, there was no difference in HbA1c between groups before supplementation, the Control (Ctrl) and SA_1.5%_ groups displayed increased (∼30%) HbA1c values at sacrifice while significant lower glycated haemoglobin was observed in the SA_15%_ group. Moreover, HbA1c values were decreased in the SA_15%_ group after supplementation compared to presupplementation values.

**Table 2:** Fasting plasma metabolites, hormones and enzyme concentrations at sacrifice (except HbA1c: pre- and postsupplementation).

	Ctrl	SA_1.5%_	SA_15%_
Presupplementation HbA1c (%)	5.3 ± 0.1	5.5 ± 0.2	5.5 ± 0.2
Postsupplementation HbA1c (%)	7.0 ± 0.3	7.0 ± 0.4	5.2 ± 0.2*†
NEFA (mM)	0.97 ± 0.05	0.92 ± 0.06	0.71 ± 0.07‡
TG (mM)	1.02 ± 0.05	1.06 ± 0.05	0.91 ± 0.06
Adiponectin (µg/ml)	7.40 ± 0.55	6.04 ± 0.51	6.66 ± 0.32
Total KB (µmol/l)	1295 ±188	1815 ± 356	2220 ± 237‡
Total cholesterol (mM/l)	2.23 ± 0.18	2.18 ± 0.11	2.91 ± 0.11*
AST (U/l)	71 ± 5	80 ± 6	98 ± 5‡
ALT (U/l)	81 ± 5	77 ± 7	84 ± 7

Data are median ± s.e.median.

KB, ketone bodies; SA, sebacic acid; TG, triglycerides.

* p value < 0.01 as compared to control group.

† Different from presupplementation.

‡ p value < 0.05.

### Oral Glucose Tolerance Test

**Figure 2:**
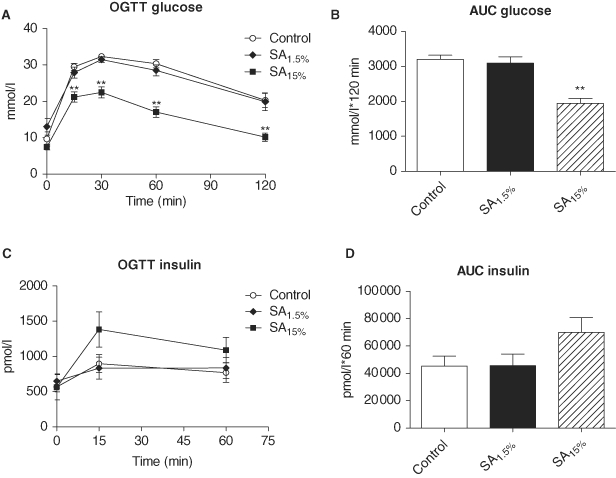
Oral glucose tolerance test measurements performed at the end of the supplementation period: (A) blood glucose, (B) area under the curve (AUC) blood glucose, (C) plasma insulin and (D) insulin AUC. Open circle: Ctrl, black diamond: SA_1.5%_ and black square: SA_15%_. ^*^p value < 0.05, ^**^p value < 0.01 as compared to control group.

To determine the effect of SA on whole-body glucose homeostasis, we examined glucose clearance during an OGTT. The SA_15%_ group displayed a significant improvement in glucose clearance (figure 2) resulting in ∼40% reduction in area under the glucose curve (p < 0.01). This effect was associated with a tendency for an improved insulin response in the SA_15%_ compared with the other groups. The AUC_ins_/AUC_glucose_ was approximately two times higher in the SA_15%_ group compared with the control group although it did not reached significance: 0.31 ± 0.1 vs. 0.14 ± 0.02 (pmol/l × 60 min) × (mmol/l × 60 min)^−1^ (p = 0.057).

### Liver Composition

As shown in Table 1, higher levels of TG were found in the liver of SA_1.5%_ (+12%) and SA_15%_ (+20%) mice compared with Ctrl, although the differences were not statistically significant (ns). Moreover, liver glycogen levels tended to be reduced in SA_1.5%_ (−25%, ns) and SA_15%_ mice (−55%, ns) compared with Ctrl. Liver cholesterol concentrations were not different between all groups.

**Table 1:** Postsupplementation (sacrifice) fasting liver TG, glycogen and cholesterol concentrations.

Liver content	Ctrl	SA_1.5%_	SA_15%_
TG (mg/g tissue)	40.1 ± 4.4	45.1 ± 2.5	47.9 ± 3.1
Glycogen (mg/g tissue)	25.0 ± 3.5	19.0 ± 2.9	11.0 ± 2.1
Cholesterol (mg/g tissue)	2.8 ± 0.1	2.7 ± 0.1	3.1 ± 0.1

Data are median ± s.e.median.

SA, sebacic acid; TG, triglycerides.

### Plasma Results at Sacrifice

As shown in Table 2, we observed significantly decreased NEFA concentrations after 15% supplementation. Neither plasma TG nor adiponectin concentrations were changed after SA supplementation. Total ketone bodies (KB) and cholesterol concentrations were significantly increased in the SA_15%_ group. Plasma ALT values were not different between groups, although AST concentrations were slightly increased in the SA_15%_, it was still in the normal range [21].

### Liver Gene Expression

In order to better understand the mechanism of action of SA, we performed a transcriptomic analysis of the liver by comparing the SA_15%_ group to the Ctrl. Relative gene expression values (SA_15%_/Ctrl) are presented as fold change expression followed by the interval of confidence at 99.9%. The cut-off of ±30% (SA_15%_/Ctrl) has been chosen based upon manufacturer's recommendations. Genes are statistically different when p < 0.001. Amongst the 24 600 genes tested, 288 were upregulated and 182 were downregulated. The complete list is available in Supporting Information (Table S1) and selected genes are presented in Table 3.

**Table 3:** Main metabolic pathways and gene expression.

Pathway	Gene name	Fold change (SA_15%_/Ctrl)
	Pck1	0.57 (0.51–0.64)*
Gluconeogenesis	Fbp1	0.73 (0.66–0.8)*
	G6pc	<0.3
Glycolysis	Gck	<0.3
	GYS2	<0.3
Glycogen	PyGL	<0.3
	Pgm	<0.3
	SCD1	1.63 (1.47–1.81)*
	FAS	<0.3
Lipogenesis	ACC	<0.3
	SREBP-1a	<0.3
	SREBP-1c	<0.3
	PPAR*γ*	1.37 (1.23–1.52)*
	FABPs	<0.3
Fatty acid transport	FATP	<0.3
	CD36	<0.3
	LPL	1.36 (1.21–1.53)*
	CPT I	<0.3
	CPT II	<0.3
	SCAD	<0.3
	MCAD	<0.3
Fatty acid oxidation	LCAD	<0.3
	VLCAD	<0.3
	Hadhb	<0.3
	Hadhsc	<0.3
	PPAR*α*	<0.3

SA, sebacic acid.

* p < 0.001 SA_15%_ is statistically different from Ctrl group.

Briefly, we observed a clear decrease in the expression of key gluconeogenic genes: Pck1 and Fbp1 [0.57 (0.51–0.63) fold and 0.73 (0.65–0.8) fold, respectively]. Glucose-6-phosphatase was not different between groups. For lipogenesis, only SCD1 and PPAR*γ* [1.6 (1.47–1.81) fold and 1.37 (1.23–1.52) fold, respectively) were upregulated in the SA_15%_ group. LPL was increased by 1.36 (1.21–1.53) fold.

Complete raw data can be consulted at http://www.ncbi.nlm.nih.gov/geo/query/acc.cgi?token=bhkzvuyouoqeczk&acc=GSE21083

## Discussion

The current study shows that 6 weeks' supplementation with the 15% energy dose of SA promotes a long-lasting stabilization of fasting glycaemia and a clear improvement of OGTT-induced glycaemia both contributing to a marked reduction of HbA1c values. At the end of the supplementation, fasting glycaemia of the SA_15%_ group was comparable to that of lean healthy db/+ animals [22], while non-supplemented animals' fasting glycaemia increased by ∼75% during the study.

Excessive HGP is a major contributor to both fasting and postprandial hyperglycaemia and is suppressed by insulin by inhibiting the expression of gluconeogenic enzymes, namely phosphoenolpyruvate carboxykinase 1 (Pck1), fructose-1,6-bisphosphatase (Fbp1) and glucose-6-phosphatase. We were able to show that a 15% supplementation with SA during 6 weeks decreased Pck1 and Fbp1 mRNA levels (−75 and −38%, respectively). In a recent study, Gomez-Valades et al. [23] partially silenced Pck1 expression by ∼50% in the liver of db/db mice and observed a reduction of fed glycaemia confirming that the downregulation of Pck1 *per se* in the liver only can be sufficient to improve glycaemia in this model of T2D. Moreover, the phenotypes observed after SA supplementation showed similarities with the phenotype observed by Gomez-Valades et al. characterized by a dramatic decrease in liver glycogen, an increase in liver TG and an improvement of glucose tolerance. Moreover, in the current experiment, expression of Fbp1 was also reduced by 38% and it has been shown in db/db mice that troglitazone could exert hypoglycaemic effects only through decreased activity of Fbp1 [24]. Moreover, key transcription factors of Pck1 and Fbp1 were also (although non-significantly) reduced—FoxO1: −30%, HNF4*α*: −20% and PGC1*α*: −25%. The role of FoxO1 in the pathophysiology of db/db mice has already been shown as well as the hypoglycaemic effect of its inhibition [25].

During OGTT, glycaemia was markedly reduced (AUC decreased by ∼40%) in the SA_15%_ group likely due to an improved (+∼30%), although non-significant, glucose-induced insulin secretion compared with both the SA_1.5%_ and control groups. Improved glucose-induced insulin secretion is probably due to decreased gluco- and lipotoxicity on the *β* cell. Indeed, beside chronically decreased glycaemia (supported by decreased HbA1c values), we observed after sacrifice that fasting plasma NEFA concentrations in the SA_15%_ group were decreased by ∼20%. Chronically lowered plasma NEFA concentrations, if confirmed, could have participated in parallel with decreased glycaemia to the improvement of glucose tolerance. Improvement of glucose tolerance could also be attributed to the relative decreased food intake observed after SA_15%_ compared with the control group. This is, however, unlikely as neither body weight nor body composition was different between groups. The improvement of diabetes symptoms in the SA_15%_ group is likely associated with a decreased loss of energy from glucose in urine. Therefore, food intake in the SA_15%_ group may reflect a more ‘normal’ situation compared with the unbalanced situation in the control group where animals lose much energy in the urine and consequently eat more to compensate. Similarly, the increased water consumption, usually observed in diabetes, was normalized in the SA_15%_ group (4 ml/day) but not in the control (12 ml/day) or low SA group (10 ml/day).

Even though not statistically significant, the 56% reduction in liver glycogen is important. Glycogen content is modulated by blood glucose concentration and by the insulin/glucagon plasma ratio. Although we did not measure glucagon, fasting insulinaemia were not different between groups therefore the chronic reduction of glycaemia was probably the primary cause of reduced liver glycogen in our experiment. On the other hand, during reduced GNG, liver glycogen could be used to maintain euglycaemia.

Although no toxicity of SA has been observed in rat and rabbit [26], the 20% increase in liver TG (although not significant) should not be overlooked. As none of the adipogenic genes expression were increased, *de novo* lipogenesis is unlikely to explain this increase. Recent papers proposed interesting hypothesis for increased liver TG through PPAR*γ* and related target genes upregulation [27,28]. Matsusue et al. [27] showed that liver overexpression of FSP27 lead to hepatic steatosis by a reduced FA oxidation and a reduced TG turnover through an unknown mechanism. In the current experiment, we also observed increased PPAR*γ* (∼1.4-fold), adipsin and genes of the CIDE family (CIDE-a and FSP27; ∼1.4- and 2.1-fold increase, respectively) expression. As liver free fatty acid (FFA) concentrations were not different between groups (data not shown) reduced FFA oxidation is unlikely; a reduced TG turnover would more likely explain relative steatosis. The increased plasma KB concentrations are difficult to interpret as plasma NEFA concentrations were decreased. This could be due to the decreased Pck1 expression through decreased cataplerosis (the mechanism by which TCA cycle intermediates are used) as shown by Hakimi et al. [29]. When Pck1 is decreased, cataplerosis is also decreased; consequently Acetyl CoA is more available for KB synthesis.

Interestingly, insulin sensitivity of rats and mice fed a high-fat diet rich in medium-chain FAs was preserved in muscles and adipose tissue but not in the liver [30]. Authors also observed an approximately six times increase in liver TG induced by MCFA. These results show that mono- or dicarboxylic medium-chain FAs are interesting substrates for reducing insulin resistance despite increased liver TG content.

In conclusion, after a 6 weeks' supplementation with SA we were able to show promising results on glucose metabolism like decreased fasting glycaemia improved HbA1c level and improved glucose tolerance in db/db mice. The mechanism of action is still unclear but SA supplementation may act synergistically at the level of glucose production by reducing hepatic glucose output, or by improving glucose-induced insulin secretion or peripheral insulin sensitivity. However, additional experiments are required to address the precise mechanism of action of SA on both glucose production and insulin sensitivity.

Finally, medium- or long-chain di-acids are a novel class of compounds that seems to present interesting properties for glucose control. Indeed, another dicarboxylic acid, norbixin (a carotenoid with 20 carbon-chain length and used as a natural pigment from the achiote shrub) has been shown to increase glucose uptake in 3T3-L1 adipocyte [31]. It would be therefore worth investigating further the promising properties of this family of compounds.

## Supporting Information

**Table S1.** Complete list of differentially regulated genes with an arbitrary cut-off at +30% (grey cells) or −30%-fold with p < 0.001. The ‘fold change’ column is the difference between the SA15% group and the control group. The third and the fourth columns represent the lower and upper confidence limits (LCL and UCL). Genes are sorted by alphabetical order.
